# Viral cGAMP nuclease reveals the essential role of DNA sensing in protection against acute lethal virus infection

**DOI:** 10.1126/sciadv.abb4565

**Published:** 2020-09-18

**Authors:** Bruno Hernáez, Graciela Alonso, Iliana Georgana, Misbah El-Jesr, Rocío Martín, Kathy H. Y. Shair, Cornelius Fischer, Sascha Sauer, Carlos Maluquer de Motes, Antonio Alcamí

**Affiliations:** 1 Centro de Biología Molecular Severo Ochoa (Consejo Superior de Investigaciones Científicas and Universidad Autónoma de Madrid), Madrid, Spain.; 2 Department of Microbial Sciences, University of Surrey, Guildford, UK.; 3 Department of Medicine, University of Cambridge, Cambridge, UK.; 4 Max Planck Institute for Molecular Genetics, 14195 Berlin, Germany.; 5 Max Delbrück Center for Molecular Medicine, Berlin Institute of Health, Berlin, Germany.

## Abstract

Cells contain numerous immune sensors to detect virus infection. The cyclic GMP-AMP (cGAMP) synthase (cGAS) recognizes cytosolic DNA and activates innate immune responses via stimulator of interferon genes (STING), but the impact of DNA sensing pathways on host protective responses has not been fully defined. We demonstrate that cGAS/STING activation is required to resist lethal poxvirus infection. We identified viral Schlafen (vSlfn) as the main STING inhibitor, and ectromelia virus was severely attenuated in the absence of vSlfn. Both vSlfn-mediated virulence and STING inhibitory activity were mapped to the recently discovered poxin cGAMP nuclease domain. Animals were protected from subcutaneous, respiratory, and intravenous infection in the absence of vSlfn, and interferon was the main antiviral protective mechanism controlled by the DNA sensing pathway. Our findings support the idea that manipulation of DNA sensing is an efficient therapeutic strategy in diseases triggered by viral infection or tissue damage–mediated release of self-DNA.

## Teaser

DNA sensing inhibition by viral cGAMP nuclease impairs anti-viral responses and protective immunity.

## INTRODUCTION

Cells constitute a hostile environment armed with multiple immune sensors that converge in the production of antiviral molecules including inflammatory cytokines and interferons (IFNs). Intracellular DNA is a potent inducer of IFN and antiviral immune responses (*1*) that signals via stimulator of interferon genes (STING) (*2*). STING is an endoplasmic reticulum–resident protein that becomes activated upon binding to cyclic dinucleotides, in particular cyclic guanosine monophosphate (GMP)–adenosine monophosphate (AMP) (cGAMP), and acts as scaffold for the recruitment of TANK-binding kinase 1 (TBK1) and IFN-responsive factor 3 (IRF3) and the induction of type I IFN (IFN-I) activation (*2*–*4*). cGAMP is produced by the cGAMP synthase (cGAS) in response to cytosolic DNA sensing of both exogenous and endogenous origin (*4*–*6*), and besides activating STING in the infected cell, can spread and activate adjacent cells via cell junctions or when packaged into viral particles (*4*, *7*). In addition to cGAS, other DNA sensors acting upstream of STING have been identified including IFI16 (*8*); the DNA damage proteins DNA-PK, MRE11, and PQBP1 (*9*–*11*); and the helicases DHX9, DDX36, and DDX41 (*12*, *13*), although the biochemical mechanisms that connect these molecules to STING and the induction of IFN-I responses are not as clear as for cGAS. Besides induction of IFN and other cytokines, the cGAS-STING pathway is also able to initiate autophagy and this has been shown to limit replication of viruses, such as the human immunodeficiency virus or herpes virus simplex 1, and intracellular bacteria, such as *Mycobacterium tuberculosis* (*14*, *15*). How this function contributes to pathogen clearance and protective responses in the infected host, though, remains to be determined.

Poxviruses are a family of successful DNA viruses that have evolved sophisticated mechanisms to deal with the host immune response (*16*). Most DNA viruses replicate their genomes in the nucleus and are therefore susceptible to predominantly nuclear sensors such as IFI16 or DNA-dependent protein kinase (DNA-PK). However, poxviruses are highly unusual viruses that replicate their DNA genomes exclusively in the cell cytosol, where the chief sensor cGAS operates. The ancestral origin of the cGAS-STING axis (*17*) and the wide range of species infected by poxviruses suggest that poxviruses must have evolved mechanisms to counteract cytosolic DNA sensing. In line with this, we provided evidence that poxviruses including vaccinia virus (VACV) and ectromelia virus (ECTV) were capable of preventing STING activation during infection and in response to exogenous DNA and cGAMP (*18*). Inhibition of STING activation was independent of the two viral inhibitors that target DNA-PK (*19*, *20*), a DNA sensor that operates via STING (*11*), and did not occur with modified VACV Ankara (MVA), an attenuated and highly immunogenic VACV strain currently used as a popular vaccine vector. Recently, Eaglesham *et al*. (*21*) found that the VACV gene *B2R* encodes the first-in-class cytosolic nuclease degrading cGAMP and therefore inhibiting STING in response to intracellular DNA. B2, which was renamed poxin, is present in most virulent orthopoxviruses, but it is absent in MVA, thus providing a potential mechanistic explanation for our previous results. Although poxin is conserved in most orthopoxviruses, it is generally not expressed as a single gene like in VACV. The orthopoxvirus poxin gene is rather fused with a second gene that has notable similarity to the short members of the Schlafen (Slfn) family of mammalian proteins, which are IFN regulated and initially reported as modulators of T cell quiescence (*22*). The Poxin-Schlafen fusion, or viral Schlafen (vSlfn), is well conserved across orthopoxviruses including the emerging zoonotic monkeypox virus or the highly virulent camelpox virus and ECTV, suggesting an important function that could modulate cGAMP nuclease activity. Unexpectedly, the entire vSlfn gene is mutated in variola virus (VARV), the causative agent of smallpox. In vivo, deletion of poxin from VACV caused only a minor effect on viral replication in the skin of mice (40-fold decrease in virus titers) and no effect on virulence was reported (*21*). These results suggest that the impact of the cGAS-cGAMP-STING pathway on protection against poxvirus infection is minor and that this pathway has little relevance. In addition, infection of transgenic mice has shown that mice lacking cGAS were slightly more susceptible to high doses of VACV (*23*). Equally, STING-deficient mice were slightly more susceptible to ECTV, but the lack of cGAS had varied results on ECTV virulence, with one study reporting a change of susceptibility (*24*) and another one reporting barely any difference (*25*).

ECTV is a mouse-specific pathogen that causes mousepox, an acute, systemic, and highly lethal disease that resembles human smallpox. Following subcutaneous infection in the footpad of susceptible mice strains, ECTV spreads through the lymphatic system to vital organs, where massive viral replication often culminates in rapid death (*26*). Here, by using virus genetics in ECTV and the infection of its natural host, we reveal the enormous contribution of the cGAS-STING pathway in conferring protection against lethal poxvirus infection. Our results show that ectopically expressed vSlfn is as active as its poxin domain and that deletion of the entire vSlfn or its poxin domain rendered ECTV unable to prevent STING, TBK1, and IRF3 activation in macrophages, implying that vSlfn is an essential viral antagonist of this pathway. ECTV lacking vSlfn or its poxin domain was markedly attenuated in several murine models of infection, and animals mounted a potent IFN response that allowed survival to a high lethal dose. Moreover, the vSlfn deletion mutant virus regained virulence in mice lacking a functional IFN system, demonstrating that IFN responses mediate the cGAS/STING-induced protection against acute viral infections. Our results therefore demonstrate the high relevance of the cGAS-STING pathway in the activation of the immune system in response to one of the most virulent poxviruses, similar to the smallpox-causing agent in humans, and the effective blockade of this pathway by a viral cGAMP nuclease.

## RESULTS

### ECTV vSlfn is a cytosolic DNA sensing inhibitor

**Fig. 1:**
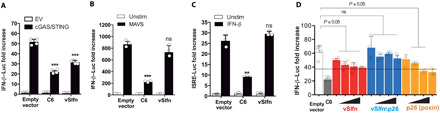
ECTV vSlfn is a modulator of the cytosolic DNA sensing pathway. (**A** to **C**) HEK293T cells were transfected with the firefly luciferase reporter gene under the (A and B) IFN-β or (C) ISRE promoter, together with vSlfn, and VACV C6 (which inhibits phosphorylation and activation of IRF3 and IRF7) or empty vector (EV) as positive and negative controls, respectively. (A) Cells were stimulated or not by simultaneous transfection of cGAS and STING, and luciferase activity was measured 16 hours later. (B) As in (A), but cells were transfected with MAVS or empty vector. (C) Cells were transfected as above but stimulated 24 hours after transfection with recombinant IFN-β. vSlfn had no effect on the modulation of IFN-β signaling. (**D**) The ability of vSlfn p26–like (orange) or Schlafen-like (blue) domains to prevent activation of the DNA sensing pathway was independently tested and compared to full-length vSlfn as in (A). Data are from one representative experiment of at least three, each performed in triplicate or quadruplicate. Data are represented as mean ± SD. *P < 0.05, **P < 0.01, or ***P < 0.001 (unpaired Student’s *t* test), compared to empty vector. ns, not significant.

Our previous results demonstrating that virulent poxviruses including ECTV prevent STING activation at a step downstream of cGAS activation (*18*) suggested the existence of one or multiple viral antagonists of this axis that prevent activation of cytosolic DNA sensing signaling during ECTV infection. Recently, cGAMP-specific nuclease activity has been assigned to VACV poxin (*21*), which is equivalent to the N-terminal baculovirus–like p26 domain from ECTV vSlfn. We therefore examined a potential role for ECTV vSlfn in the modulation of cytosolic DNA sensing pathway. Using a luciferase-based cellular assay in human embryonic kidney (HEK) 293T cells, a cell line in which this pathway can be functionally restored by cotransfection of expression vectors for cGAS and STING (*5*), vSlfn significantly reduced activation of an IFN-β promoter controlling luciferase reporter triggered by cGAS and STING (Fig. 1A). This inhibition was specific to cGAS/STING: vSlfn had no effect on IFN-β activation after stimulation by the central RNA sensing adaptor mitochondrial antiviral signaling (MAVS) (Fig. 1B) or after infection with the RNA virus Sendai virus (fig. S1A). vSlfn expression was also unable to block the activation of IFN-stimulated response elements (ISREs) mediated by IFN-β (Fig. 1C) or the activation of nuclear factor κB (NF-κB)–driven luciferase reporter upon exposure to interleukin-1β (IL-1β) or expression of the tumor necrosis factor (TNF) receptor–associated factor 6 (TRAF6) (fig. S1, B and C).

In most orthopoxviruses, vSlfn is composed of two domains with different evolutionary origin. To further discriminate the contribution to cytosolic DNA sensing inhibition of the two different domains in ECTV vSlfn, we next cloned them separately (fig. S1D): residues 1 to 186 encoding the N-terminal baculovirus–like p26 domain (recently renamed poxin) and residues 196 to 503 encoding the C-terminal domain, which resembles the short members of the murine family of Slfn proteins (*27*). Expression of the poxin/p26 domain, but not the Slfn-like domain, was sufficient to suppress cGAS/STING signaling. In addition, the extent of inhibition observed in the presence of poxin/p26 was similar to that observed with the intact vSlfn protein (Fig. 1D), indicating that the ECTV poxin/p26 domain retains the full capability of vSlfn to prevent activation of DNA sensing through the cGAS/STING axis despite its fusion to the Slfn-like domain.

### vSlfn efficiently counteracts the cGAS/STING pathway during infection

**Fig. 2:**
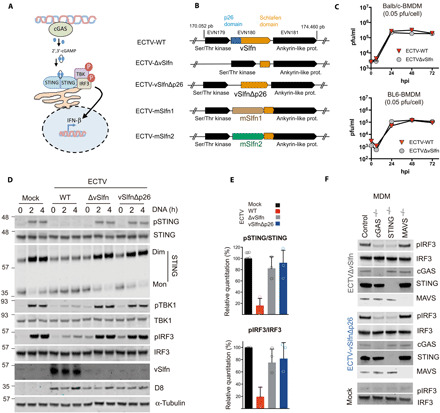
vSlfn inhibits the cGAS-STING axis in ECTV infections. (**A**) Schematic representation of the cGAS/cGAMP/STING pathway. Upon DNA recognition, cGAS synthesizes 2′,3′-cGAMP and activates STING, which, in turn, leads to IRF3 nuclear translocation and the expression of cytokine genes such as IFN-β. (**B**) Genomic organization of vSlfn and surrounding loci. vSlfn (*EVN180* in ECTV Naval strain) contains an N-terminal p26 domain (in blue) and an Slfn-like domain (in orange) and is surrounded by a Ser/Thr kinase (*EVN179*) and an ankyrin-like protein gene (*EVN181*) shown in black. mSlfn1 and mSlfn2 refer to murine Slfn genes 1 and 2 replacing vSlfn. (**C**) ECTV growth curves in the presence or absence of vSlfn in bone marrow–derived macrophages (BMDMs) from Balb/c or BL6 mice. BMDMs were infected with 0.05 plaque-forming units (pfu)/cell of ECTV wild type (ECTV-WT) or ECTV lacking vSlfn (ECTV∆vSlfn), and viral titers were determined at indicated times. (**D**) Infection with ECTV∆vSlfn or ECTV lacking the vSlfn p26–like domain (ECTV-vSlfn∆p26) failed to suppress STING phosphorylation and dimerization as well as TBK1 and IRF3 phosphorylation in response to transfected DNA in monocyte-derived macrophages (MDMs). Cells were infected with 2 pfu/cell of the indicated viruses for 6 hours and subsequently stimulated with exogenous DNA for increasing times, before being analyzed by SDS–polyacrylamide gel electrophoresis (PAGE) for the indicated proteins. D8 is an unrelated ECTV protein used as infection marker. (**E**) STING and IRF3 phosphorylation levels from three independent experiments performed as above. (**F**) IRF3 phosphorylation in response to ECTV∆vSlfn or ECTV-vSlfn∆p26 infection required cGAS and STING, but not MAVS. MDMs deficient for the indicated proteins were infected with 5 pfu/cell of the indicated viruses for 6 hours before being analyzed by SDS-PAGE for the indicated proteins. All data are representative of at least three experiments.

To validate the ability of vSlfn to antagonize cGAS/STING signaling in virus-infected cells, we next generated a set of recombinant ECTVs deficient for vSlfn (ECTVΔvSlfn) or its p26 domain (ECTV-vSlfnΔp26) (Fig. 2B). To ensure that the phenotype of these viruses is not due to the insertion of a marker gene affecting the expression of neighboring genes or the selection of inadvertent mutations in other genes, the puromycin resistance and enhanced green fluorescence protein (EGFP) marker genes used to isolate recombinant viruses were removed by recombination and the viral genomes were fully sequenced. We additionally confirmed the absence of vSlfn expression in ECTV∆vSlfn-infected cell extracts by immunoblotting using vSlfn-specific serum detecting the p26 domain (fig. S2A) as well as the expression of the C-terminal Slfn–like domain in ECTV-vSlfnΔp26–infected cell extracts using Strep-tag immunoblotting (fig. S2B). We also demonstrated that vSlfn is not essential for replication in cell culture because both viruses replicated with kinetics similar to that of wild-type ECTV (ECTV-WT) in multiple cell lines including murine bone marrow–derived macrophages (BMDMs) (Fig. 2C and fig. S3A*)*. We then addressed the ability of these viruses to suppress DNA sensing in monocyte-derived macrophages (MDMs), which, unlike HEK293T cells, express most cytosolic DNA sensors and respond to exogenous DNA. As we have previously shown (*18*), ECTV-WT efficiently inhibited DNA-induced activation of STING and IRF3. Conversely, ECTVΔvSlfn and ECTV-vSlfnΔp26 were completely impaired for such inhibition because infection with both mutant viruses showed notably high levels of STING, TBK1, and IRF3 phosphorylation and STING dimerization, indicative of activation of the cGAS-STING axis (Fig. 2, A and D). Similar results were observed when infected MDMs were stimulated with cGAMP (fig. S4), consistent with the reported cGAMP nuclease activity. Notably, the extent of DNA-induced activation observed for STING, TBK1, and IRF3 during ECTVΔvSlfn and ECTV-vSlfnΔp26 infection was similar to that found in mock-infected cells, indicating that vSlfn is a critical inhibitor of cytosolic DNA sensing in ECTV (Fig. 2D).

In these time-course challenge experiments, we noticed that MDM infected with ECTVΔvSlfn or ECTV-vSlfnΔp26 (but not challenged with DNA or cGAMP) showed increased levels of pIRF3 compared to ECTV-WT infection. This suggested that infection with vSlfn mutant viruses was sufficient to trigger IRF3 activation. To confirm this as well as the specificity of this IRF3 response, MDMs deficient for cGAS, STING, or MAVS were infected with our set of viruses at higher multiplicity of infection (MOI). In agreement with our previous observations, ECTVΔvSlfn or ECTV-vSlfnΔp26 infection triggered high levels of IRF3 phosphorylation and these were abolished in the absence of cGAS and STING, but not MAVS (Fig. 2F). Despite the inability of viruses lacking vSlfn to counteract cGAS/STING-dependent activation of IRF3 during infection, the replication kinetics of these viruses remained undistinguishable in MDM lacking cGAS or STING (fig. S3B). Together, these results defined vSlfn p26 domain as a potent inhibitor of the cGAS-STING signaling during ECTV infection.

### ECTV vSlfn strongly contributes to virulence in the poxvirus natural route of infection

**Fig. 3:**
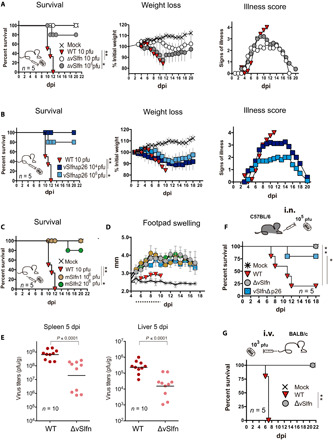
vSlfn is an essential virulence factor during mousepox infection. (**A**) Mousepox pathogenesis and survival were analyzed after subcutaneous footpad infection of Balb/c mice with increasing doses of ECTV-WT or ECTV∆vSlfn (∆vSlfn). For better clarity, only mortality, weight loss, and signs of illness corresponding to the 10 and 10^6^ pfu doses are shown. (**B**) Subcutaneous footpad infection of mice with ECTV-vSlfn∆p26 (vSlfn∆p26) was analyzed as before. (**C**) Survival after replacement of vSlfn with its murine homologs mSlfn1 and mSlfn2 was evaluated after infection with 10^6^ pfu of ECTV-mSlfn1 and ECTV-mSlfn2, respectively. (**D**) Size of the footpad (mm) of mice inoculated with 10^6^ pfu of the indicated viruses is expressed as mean ± SEM. Dotted line indicates time points at which significant differences [multiple *t* tests with false discovery rate (FDR) = 1%, *P* < 0.01] were observed between WT and mutant ECTVs. Weight data are expressed as the mean ± SEM of the five animal weights compared to their original weight at the day of inoculation, and signs of illness as a score ranging from 1 to 4. (**E**) Virus titers in major target organs at 5 dpi after subcutaneous footpad infection of Balb/c mice with 10^3^ pfu of ECTV∆vSlfn or ECTV-WT (Mann-Whitney test). Detection limit of the assay was 10^2^ pfu/g. *n* = 10. (**F**) Survival of C57BL/6 mice inoculated intranasally (i.n.) with 10^5^ pfu of the indicated viruses. (**G**) Survival of Balb/c mice after intravenous (i.v.) inoculation by injection in the tail vein of 10^3^ pfu of the indicated viruses. For survival data, *P < 0.05 and **P < 0.005 (Mantel-Cox test). A representative experiment of at least two performed is shown in every case. See table S1 for complete survival data.

Mousepox disease is an excellent model to study poxvirus pathology and the host response to viruses that cause acute lethal infection and/or spread via a lympho-hematogenous route (*26*, *28*, *29*). To elucidate the impact of cytosolic DNA sensing activation on the course of lethal mousepox disease, we took advantage of ECTV∆vSlfn, a virus unable to block the cGAS-STING-IRF3 axis, and determined its virulence. Thus, susceptible Balb/c mice were infected with increasing doses of ECTV∆vSlfn [ranging from 10 to 10^6^ plaque-forming units (pfu)] by subcutaneous inoculation in the footpad, which approximates the natural route of infection. We observed a marked virus attenuation in lethal dose of 5 logs compared to ECTV-WT infection (Fig. 3A and table S1). In the case of ECTV-WT, all the animals succumbed to infection with just 10 pfu before 12 days post-infection (dpi), whereas only one mouse succumbed to infection with 10^6^ infectious units of ECTVΔvSlfn, the maximum dose that our virus stocks permitted. Although ECTV∆vSlfn-infected animals developed severe signs of mousepox disease that peaked around 3.2 dpi and suffered significant weight loss, they completely recovered from illness by 16 to 20 dpi. In line with results from in vitro assays, a similar degree of attenuation was observed after subcutaneous inoculation of increasing doses of ECTV-vSlfnΔp26 (Fig. 3B and table S1). The same result was obtained after infection with two additional recombinant ECTVs, where vSlfn coding sequence was replaced by the short members of the murine *Slfn* family lacking the p26 domain: *mSlfn1* (ECTV-mSlfn1) and *mSlfn2* (ECTV-mSlfn2). Only one animal died after infection with the highest dose assayed of ECTV-mSlfn2 (10^6^ pfu), while the rest of animals survived the disease (Fig. 3C and table S1), indicating that mSlfn1 and mSlfn2 do not complement vSlfn during ECTV infection and suggesting that the strong attenuation observed is mostly due to activation of the cGAS-STING axis in the absence of the p26 domain.

Following footpad infection of susceptible mice by subcutaneous inoculation, ECTV spreads to the draining popliteal lymph node (DPLN), where it replicates. Then, at 2 to 3 dpi, ECTV spreads via efferent lymphatics to bloodstream to reach major target organs, such as spleen and liver, where massive replication usually occurs leading to animal death (*26*). In every case where ECTV infection proceeded in the absence of the p26 domain, the attenuation was accompanied by a clear and significant increase in footpad swelling from 5 dpi, compared to ECTV-WT infection (Fig. 3D). Despite this potent inflammatory response elicited at the inoculation site, ECTV∆vSlfn reached the major target organs, spleen and liver. However, virus load determination revealed a nearly 2-log significant decrease in viral yields from both organs at 5 dpi, which allowed animal survival in the absence of vSlfn (Fig. 3E).

### ECTV vSlfn contributes to virulence in experimental routes of infection

ECTV is naturally transmitted by direct contact, which can be mimicked by footpad inoculation. Nevertheless, other routes of infection, involving aerosols or droplets, are possible to reproduce experimental, systemic, and lethal mousepox (*26*). To ascertain whether repression of the cGAS-STING axis is required for a successful infection independently of the virus administration route, we assayed intranasal inoculation in C57BL/6 mice, a strain that is resistant to mousepox after footpad infection but becomes susceptible to ECTV infection after intranasal inoculation with high doses. Once again, the infection with either the vSlfn deletion mutant virus or ECTV-vSlfn∆p26 resulted in reduced mortality compared to ECTV-WT infection (Fig. 3F). We next assayed infection by intravenous inoculation, which bypasses the DPLN, allowing direct ECTV spreading to major target organs. Even in these conditions, ECTV∆vSlfn exhibited a major attenuated phenotype (Fig. 3G), highlighting vSlfn as an essential virulence factor independently of the administration route, which operates as a potent mechanism to prevent cGAMP-mediated STING activation enabling virus dissemination during infection.

### Exogenous cGAMP modifies mousepox outcome

**Fig. 4:**
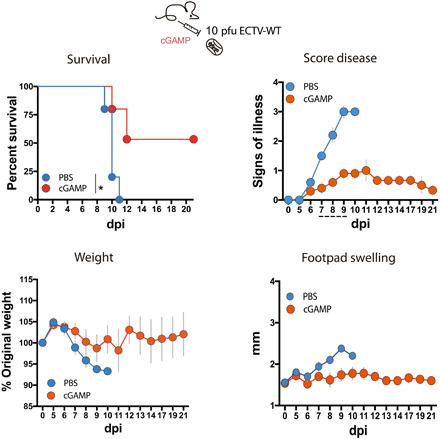
cGAMP effect on mousepox disease. A single dose of 140 μg of 2′,3′-cGAMP per animal or phosphate-buffered saline (PBS) was injected into the footpad of Balb/c mice 16 hours before subcutaneous infection with 10 pfu of ECTV-WT in the footpad. Signs of illness ranging from 1 to 4 and footpad swelling (millimeter) are shown as mean ± SEM. A representative experiment of two performed is shown with five animals per experimental condition. For survival data, *P < 0.05 (Mantel-Cox test), while dotted line indicates those time points at which significant differences (multiple *t* tests with FDR = 1%, *P* < 0.01) in score disease were observed.

Last, we explored the impact on disease outcome of exogenous administration of the second messenger cGAMP, which is a STING activator generated after cGAS recognition of cytosolic DNA. To this effect, a single dose of 140 μg of cGAMP per animal was locally administered at the inoculation site 16 hours before subcutaneous inoculation of ECTV in the footpad. This cGAMP injection was sufficient to reduce and delay mortality associated to a lethal dose of ECTV (Fig. 4). cGAMP-injected animals also exhibited reduced weight loss and delayed signs of mousepox, conferring cGAMP a protective role. These results revealed the critical role of STING-dependent DNA sensing in host protection against lethal poxvirus infection.

### IFN-I mediates protection in the absence of vSlfn

**Fig. 5:**
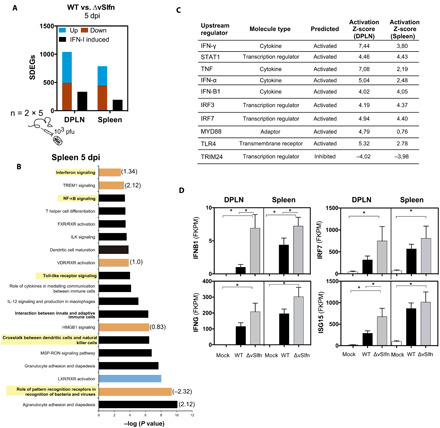
The absence of vSlfn upon infection elicits an IFN-based host response. (**A** to **D**) Two groups of five Balb/c mice were mock-infected or infected with 1000 pfu of ECTV-WT or ECTV∆vSlfn. Differential RNA-seq analysis of the host response comparing ECTV-WT versus ECTV∆vSlfn infection was performed in DPLN and spleen from two groups of five infected at 5 dpi. (A) Significant differentially expressed genes (SDEGs) identified after Cufflinks analyses (fold change > 2 and *P* < 0.005) were classified as up-regulated (blue) or down-regulated (red), while black bars denote those up-regulated genes identified as IFN-I–induced genes by Interferome. (B) Corresponding pathway analyses showing the enrichment of innate immunity pathways (highlighted). Z-score values for activated (orange) or repressed (blue) predicted pathways are indicated. (C) The Ingenuity Pathway Analysis (IPA) upstream regulator analysis identified IFN-I–related genes as upstream regulator molecules responsible for the changes in gene expression in the absence of vSlfn. (D) Expression values of the indicated IFN-I–related genes are expressed as the mean from two groups with five mice each ± SEM. FPKM, fragments per kilobase per million reads mapped. *P < 0.005 (Cufflinks differential expression test).

To elucidate the nature of the protective host response elicited after activation of the cGAS-STING axis upon ECTV∆vSlfn infection, we carried out transcriptomic analysis [RNA sequencing (RNA-seq)] from relevant infected tissues. Differential gene expression analyses retrieved a large number of statistically differentially expressed genes (SDEGs) after comparison with wild type infection in the DPLN and spleen at 5 dpi (Fig. 5A). A prominent proportion of these SDEGs was identified as IFN-I induced by Interferome v2.1 database, 32.5 and 28.2% in DPLN and spleen, respectively. IFN-β (*IFNb1*) and IFN-γ, as well as diverse interferon-stimulated genes (ISGs), such as *IRF7* or IFN-stimulated gene 15 (*ISG15*), were found to be up-regulated in both DPLN and spleen at 5 dpi in the absence of vSlfn (Fig. 5D). Accordingly, diverse cellular pathways related to innate immunity were found enriched by these SDEGs and predicted to be active at 5 dpi in these tissues. Among these, we found “Role of pattern recognition receptors in virus recognition,” “IFN signaling,” “NF-κB signaling,” or “Toll-like receptor signaling” pathways. The upstream regulator analysis identified several relevant molecules of the IFN response with positive Z-scores, such as *STAT1*, *TLR4*, *IFN-*α, *IRF3*, or *IRF7*, as responsible for the overall changes. On the contrary, an inhibited state was predicted for the negative regulator of the IFN signaling TRIM24 (Fig. 5, B and C).

**Fig. 6:**
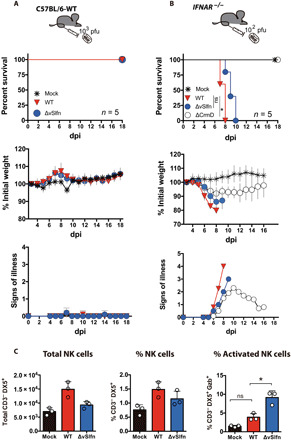
ECTV∆vSlfn virulence in mice lacking IFN-I signaling. (**A**) C57BL/6 WT mice were resistant to subcutaneous inoculation in the footpad with 10^3^ pfu of ECTV∆vSlfn or ECTV-WT. (**B**) ECTV∆vSlfn regained virulence after subcutaneous infection in the footpad of mice deficient in IFN-I signaling (*IFNAR*^−/−^), while a mutant virus lacking CrmD (∆CrmD) retained an attenuated phenotype. In both cases, weight loss is expressed as mean ± SEM of the initial weight and signs of disease. A representative experiment of two is shown in every case. For survival data, *P < 0.05 (Mantel-Cox test), while dotted line indicates those time points at which significant differences (multiple *t* tests with FDR = 1%, *P* < 0.01) in score disease were observed between ECTV-WT and ECTV∆vSlfn. (**C**) Examination by flow cytometry of cell populations in the DPLN after subcutaneous infection in the footpad of Balb/c mice revealed an increase in NK cell activation at 2 dpi in the DPLN after ECTV∆vSlfn infection, as compared to ECTV-WT infection. Three groups containing four DPLNs each were analyzed for each experimental condition. *P < 0.05 [analysis of variance (ANOVA) with Bonferroni multiple comparison test].

To verify the major role of IFN-I in the ECTV∆vSlfn-elicited protective response, we next infected C57BL/6 mice deficient in subunit 1 of the IFN-I cellular receptor (*IFNAR*^−/−^ mice), a scenario where IFN-I signaling is impeded. This absence of functional IFN-I signaling completely abolished the inherent resistance of C57BL/6 mice to footpad injection with ECTV-WT, because *IFNAR*^−/−^ animals succumbed to infection at 8 dpi (Fig. 6, A and B). C57BL/6 mice were also resistant to ECTVΔvSlfn, but when *IFNAR*^−/−^ mice were infected with ECTVΔvSlfn, a high virulence was observed and all animals died at 10 dpi, just 1 to 2 days after ECTV-WT infection (Fig. 6B). On the contrary, infection with ECTV∆CrmD, a virus lacking a viral antagonist of TNF signaling (*29*), still exhibited an attenuated phenotype in *IFNAR*^−/−^ mice (Fig. 6B), demonstrating that these animals were still capable of mounting protective responses, and ECTV needs to block the TNF-mediated pathway to become virulent in IFNAR^−/−^ mice. Therefore, in the absence of IFN signaling, the absence of vSlfn does not affect virulence, whereas the absence of CrmD attenuates the virus. These results indicate that no mechanisms other than IFN mediate protection in the absence of vSlfn and are in line with the known cross-talk between IFN-I and NF-κB pathways (*30*) and explain why pathogens have evolved antagonists of both pathways.

Last, we investigated the impact of cytosolic DNA sensing on the natural killer (NK) cell response, a major mechanism of innate immunity previously identified as essential to survive lethal mousepox (*31*) that was differentially up-regulated in the DPLNs of ECTVΔvSlfn-infected animals (fig. S5). We found an increased proportion of activated NK cells (CD3^−^, DX5^+^, Gzb^+^) in the DPLN 2 days after ECTV∆vSlfn infection compared to ECTV-WT infection (Fig. 6C and fig. S6), suggesting an effective role for these cells in the protection from fatal mousepox in the absence of vSlfn. Collectively, these results provided a direct link between cytosolic DNA recognition and a protective IFN-based response through STING activation in the infected host.

## DISCUSSION

All viruses need to overcome a wide range of cellular immune sensors to deploy and replicate their genome and successfully transmit. DNA is a potent agonist of immune responses that can be sensed by a variety of molecules, most of which converge on STING and activate IRF3. How viruses counteract DNA sensing and how DNA sensing contributes to the final outcome of the infection are not always clear. Poxviruses are the only DNA viruses that replicate their genome exclusively in the cell cytoplasm, and they were recently found to encode a viral cGAMP nuclease or poxin (*21*). Here, we have used the highly specific ECTV and its matching host species to reveal that the poxviral cGAMP nuclease is the major antagonist of IRF3 activation during infection and a critical virulence factor, and that, in its absence, the host unleashes a potent IFN response that protects animals from otherwise lethal outcome. Like in most orthopoxviruses, ECTV poxin is fused to an Slfn domain forming vSlfn. Our data showed that ECTV lacking vSlfn or the poxin domain triggered robust IRF3 activation and this required cGAS and STING. In addition, in the absence of vSlfn or poxin, ECTV was unable to counteract IRF3 activation in response to exogenous DNA or cGAMP. These results are remarkable because poxviruses have coding capacity for >200 proteins, of which ~100 are immunomodulatory (*16*), and many of these have largely redundant functions as fail-safe mechanisms. vSlfn/Poxin is thus unique in counteracting the cGAS-STING axis. Some of these fail-safe mechanisms may, however, be responsible for blocking IRF3-dependent antiviral effects because the inability of the vSlfn mutant viruses to prevent IRF3 activation triggered by viral infection did not have a detrimental impact on the ability of these viruses to replicate even in cells competent for DNA sensing signaling. Previous work by Meade *et al.* (*32*) has shown that protein F17 is also able to counteract this axis by indirectly triggering Akt-dependent degradation of cGAS. Because F17 is expressed late during infection, it is likely that both mechanisms cooperate to prevent poxvirus sensing and ensure complete suppression of antiviral responses.

Despite the presence of multiple viral inhibitors acting cooperatively, the single deletion of vSlfn/Poxin had a profound impact on virulence and resulted in a marked attenuation of infection, with animals mounting a potent IFN response that allowed survival to up to a 100,000× lethal dose. Such attenuation has only been described in virology for ECTV mutants lacking secreted IFN-I and TNF decoy receptors (*28*, *29*, *33*). The similar marked attenuation observed when deleting viral inhibitors of either IFN-I induction (vSlfn) or IFN-I activity (IFN-I decoy receptor) is consistent with the concept that the main antiviral protective mechanism controlled by the DNA sensing pathway in the infected host is the activation of IFN-I. Unleashed cGAS-STING activation during ECTVΔvSlfn infection resulted in strong inflammation in the inoculation site that correlated with a prominent IFN-I signature in the lymph node and the spleen. As a consequence, viral titers were reduced in targets organs such as liver and spleen and animals recovered from infection. A similar course of events has recently been reported using cGAS^−/−^ mice infected with ECTV (*24*). The strong activation of the cGAS-STING axis also resulted in an increased number of total and activated NK cells, which are a well-established effector mechanism against lethal mousepox infection. Collectively, these results indicate that the early recognition of ECTV via cGAS is crucial in mounting an antiviral response that limits viral dissemination, allowing the activation of protective effector mechanisms. This early antiviral response relies on IFN-I signaling because, exclusively, *IFNAR*^−/−^ mice succumbed to infection with ECTVΔvSlfn at similar times as ECTV-WT. Therefore, although autophagy induced by the cGAS-STING pathway has been shown to limit viral and bacterial replication in cell culture (*14*, *15*), the major protective mechanism induced by cGAS in the control of ECTV in the infected host is the IFN-I induction.

The fact that poxviruses have evolved a molecule to target cGAMP highlights the importance of this second messenger in immune activation. In agreement with this, administration of exogenous cGAMP at the inoculation site in the footpad protected animals from an otherwise lethal outcome, presumably by priming skin and immune-associated cells for IFN-I responses. In line with our results, cGAMP administration rescued cGAS-deficient mice from ECTV infection and this was shown to be dependent on IFN-I signaling (*24*). Therefore, production of cGAMP is a vital function of cGAS to elicit protective responses. In response to this, poxviruses evolved vSlfn/Poxin as a cGAMP nuclease. As an early gene, vSlfn/Poxin is expressed from within the viral core before genome release and genome replication occurs, allowing suppression of DNA sensing mediated by the potential recognition of the incoming virus genome by cGAS. Although it cannot be formally ruled out, our results in ECTV suggest that no upstream viral inhibitors exist and that vSlfn/Poxin has nonredundant functions, at least early during infection. This ECTV strategy of targeting cGAMP is unique among mammalian viruses and contrasts with the larger number of mammalian viruses that target cGAS and/or STING instead (*34*). This difference may reflect the unique evolutionary niche occupied by poxviruses as DNA viruses replicating in the cytosol.

The fusion of an Slfn domain, whose immunological function has not been fully elucidated (*22*), to poxin is intriguing and suggests a role of Slfn in the regulation of DNA sensing pathways. However, our in vitro results expressing vSlfn or its domains separately and the fact that ECTV-vSlfnΔp26 exhibited a similar degree of attenuation than ECTV∆vSlfn do not support a role for Slfn in STING-dependent IRF3 activation and/or virulence, although further experimentation is needed to ascertain this. Arguing against a redundant role for Slfn is the fact that although poxin is conserved in most orthopoxviruses (*21*), only those that are currently found in rodents in the wild encode a mammalian Slfn-like domain fused to it. A single poxin gene is also present in viruses evolutionary distant to the orthopoxviruses such as insect or bat poxviruses (*21*), suggesting that an ancestral vertebrate poxvirus carrying poxin evolved vSlfn as a poxin-Slfn fusion when adapting to infect rodents. This rodent poxvirus evolved to infect several other mammalian species forming what is now known as the *Orthopoxvirus* genus. Orthopoxviruses that are no longer found in rodents, such as the extinct horsepox, VARV, or VACV, show inactivating mutations in the Slfn domain (also known as gene *B3R* in these viruses), suggesting that a posterior loss of the Slfn domain may correlate with adaptation to other hosts, including humans. Unexpectedly, VARV, the smallpox agent and a human-specific virus, contains inactivating mutations not only in the Slfn domain but also in poxin. This enigmatic finding suggests that alternative strategies to effectively overcome cytosolic DNA sensing may exist in VARV; otherwise, the absence of poxin might account, at least in part, for the strong proinflammatory cytokine response during smallpox disease.

cGAS is activated not only by microbial DNA but also by binding to self-DNA released to cytosol, e.g., by nuclear or mitochondrial leakage after tissue damage, cellular stress, or genetic factors (*35*). Thus, DNA sensing through cGAS-STING has emerged as a triggering factor in the pathogenesis of type I interferonopathies and self-DNA inflammatory diseases, such as Aicardi-Goutières syndrome or systemic lupus erythematosus, and also with implications in cancer immunosurveillance (*36*, *37*). For this reason, cGAS arises as a therapeutic target to repress the DNA-induced IFN expression in these human disorders. By high-throughput screening, diverse cGAS-specific small-molecule inhibitors have been recently identified and demonstrated to have a negative impact on IFN-I expression in macrophages (*38*). The marked in vivo effect of the ECTV vSlfn domain p26 (poxin) as a potent inhibitor of innate immunity we report in this study highlights the potential of unleashing DNA sensing pharmacologically to boost antiviral protective responses and supports the therapeutic value of DNA sensing antagonists to prevent diseases triggered by self-DNA.

## MATERIALS AND METHODS

### Cells and viruses

BSC-1 European Collection of Authenticated Cell Cultures (ECACC), HEK293T (ECACC), and Raw 264.7 (ECACC) cells were maintained in 10% fetal bovine serum (FBS) containing Dulbecco’s modified Eagle’s medium (DMEM). THP-1 cells were maintained in 15% FBS–RPMI 1640 supplemented with penicillin (100 U/ml) and streptomycin (100 μg/ml) (Life Technologies). THP-1 cells knocked out for STING or MAVS have been previously described and characterized (*39*, *40*). THP-1 cells knocked out for cGAS were from InvivoGen. To obtain BMDMs, cell suspensions from femurs of mice were plated in RPMI 1640 medium supplemented with 10% FBS and 10% supernatant from L929 cells, with medium replacement every 2 days. On day 8 after culture, cells were evaluated for surface expression of cellular markers to ensure 70 to 90% of F4/80^+^ and CD11b^+^ cells.

BSC-1 cells were used to generate and propagate ECTVs. A plaque-purified and fully sequenced ECTV Naval isolate was used, and ECTV-∆CrmD has been previously described (*29*). Viral stocks were routinely tested for the absence of mycoplasma, and minimal concentrations of endotoxin levels were detected. Viral stocks were titrated twice by plaque assay in monolayers of BSC-1 cells before animal infections. Cells were incubated in semisolid carboxymethylcellulose medium with 2.5% fetal calf serum (FCS) and fixed in 10% formaldehyde at 6 dpi, and plaques were stained with 0.1% (w/v) crystal violet. Sendai virus strain Cantell was obtained from G. Towers (University College London, UK) and was used at a final concentration of approximately 50 hemagglutination units/ml.

### Construction of recombinant poxviruses

Recombinant ECTVs were generated using a transient dominant selection procedure based on puromycin resistance to avoid the presence of foreign DNA sequences in the genome of the final recombinant ECTV. Briefly, to generate ECTV∆Slfn, ECTV-vSlfn∆p26, ECTV-mSlfn1, and ECTV-mSlfn2, BSC-1 cells were infected with 0.01 pfu/cell of the ECTV Naval strain and, at 1 hour post infection (hpi), transfected with p33, pBH4, p35, and pBH2 plasmids, respectively, using FuGENE HD (Promega), following the manufacturer’s indications. These plasmids are based on pMS30 and contain the fragments corresponding to 5′ and 3′ flanking regions of *EVN180* gene (Fig. 2B), together with a downstream cassette including the EGFP marker under the control of an orthopoxvirus early/late synthetic promoter and the puromycin resistance gene. In the case of recombinant plasmids p35 and pBH2, the complementary DNAs (cDNAs) encoding mSlfn1 and mSlfn2 were polymerase chain reaction (PCR)–amplified using total murine cDNA as template with specific oligonucleotides (table S2) and respectively inserted into the restriction site between the 5′ and 3′ flanking regions of EVN180 gene. In the same way, ECTV-vSlfnStrepTag and ECTV-vSlfn∆p26StrepTag were generated using pBH5 plasmid (table S2) and ECTV Naval strain or ECTV-vSlfn∆p26 as parental viruses, respectively. When cytopathic effect was complete, cells were harvested and used as inoculum in five consecutive rounds of infection in the presence of puromycin (10 μg/ml) (Sigma-Aldrich) monitoring EGFP expression. Recombinant ECTVs were finally isolated by three successive plaque purification steps of white plaque selection in the absence of puromycin. The genome from every recombinant ECTV generated was fully sequenced using Illumina sequencing technology, and the genetic structure of the viruses and the absence of inadvertent mutations that may have been introduced during the generation of recombinant viruses were confirmed. With this information, a revertant virus was not required and the number of mice in the experiments could be reduced. Raw reads obtained after sequencing were deposited at European Nucleotide Archive (ENA), available under project number PRJEB34111.

### Virus growth curves

BSC-1 cells were infected at 37°C at high MOI (5 pfu/cell) or low MOI (0.01 pfu/cell) in the one-step or multiple-step growth curves, respectively. THP-1, BMDMs, and Raw 264.7 were infected with 0.01 or 0.05 pfu/cell at 37°C. Cells were washed after 1 hour, and fresh medium was added. At indicated times after infection, cells were harvested in their own medium, centrifuged at 1800*g* for 5 min, and resuspended in 0.5 ml of fresh medium. In all cases, samples were frozen, thawed three times, and titrated on BSC-1 cells in duplicate as described above.

### Expression plasmids

The vSlfn sequence from ECTV Naval strain was codon-optimized for expression in murine cells and obtained from GeneArt (Thermo Fisher Scientific) as a fusion with C-terminal 3xFLAG tag, and subcloned into pcDNA3.1 (Invitrogen) to generate the expression vector pvSlfn-FLAG. To generate plasmid pvSlfn∆p26-FLAG, the fragment encoding the p26 domain in the vSlfn sequence (amino acids 1 to 186) was removed from pvSlfn-FLAG. To generate pvSlfnp26-FLAG, the fragment corresponding to amino acids 196 to 503 was removed from pvSlfn-FLAG. In both cases, modifications were performed using an In-Fusion HD Cloning kit (Takara) after PCR amplification with Kapa Hifi polymerase (Kapa Biosystems) and specific oligonucleotides (table S2) using pvSlfn-FLAG (pBH21) as template.

### Reporter gene assays

HEK293T cells were seeded in 96-well plates and transfected with the indicated reporters and expression vectors using polyethylenimine. The reporter plasmids have been previously used (*41*). pIFN-β-Luc, pNF-κB-Luc, and pISRE-Luc expressed firefly luciferase (FLuc) under the control of the indicated promoters and were used at 70 ng per well. pTK-RLuc expressed *Renilla reniformis* luciferase (RLuc) and was used at 10 ng per well. For coexpression stimulation, the cells were simultaneously transfected with expression constructs for cGAS (20 ng per well), STING (20 ng per well), MAVS (40 ng per well), or TRAF6 (5 ng per well), which have been previously described (*41*) and harvested 16 hours later by washing the cells with 200 μl of ice-cold phosphate-buffered saline (PBS) and lysis in 100 μl of passive lysis buffer (Promega). Sendai virus was used 24 hours after transfection of plasmids to infect for a further 24 hours, after which cells were harvested as before. IFN-β and IL-1β were used 24 hours after transfection of plasmids to stimulate for a further 8 hours, after which cells were harvested as before. Luciferase activity was measured in a CLARIOstar plate reader (BMG Biotech), and FLuc and RLuc ratios were calculated. Data were normalized to empty vector (EV)–transfected samples and presented as fold increase values.

### DNA and cGAMP activation assays

THP-1 cells were differentiated to MDMs in the presence of phorbol 12-myristate 13-acetate (20 ng/ml) (Santa Cruz Biotechnology) for 48 hours and subsequently infected with the indicated viruses in fresh 2% FCS medium for 6 hours at MOI of 2 pfu/cell unless otherwise indicated in the figure legends. The cells were then transfected with herring testes DNA (2 μg/ml) (Sigma-Aldrich) using TransIT-LT1 (Mirus Bio) following the manufacturer’s instructions or exposed to 2′,3′-cGAMP (15 μg/ml) (InvivoGen) for the indicated lengths of time. Cells were washed in ice-cold PBS and lysed in 50 mM tris (pH 8.0), 150 mM NaCl, 1% NP-40, 0.5% sodium deoxycholate, and 0.1% SDS supplemented with benzonase (5 U/ml) (Sigma-Aldrich) as well as protease and phosphatase inhibitors (Roche). Lysates were incubated on ice for 30 min and eventually denatured at 95°C for 5 min in the presence of loading buffer. To detect STING dimerization, lysates were kept undenatured in the presence of NuPAGE LDS sample buffer (Bio-Rad) as previously described (*18*).

### SDS-PAGE and Western blotting

Whole-cell lysates were resolved by SDS-PAGE (polyacrylamide gel electrophoresis) and transferred to nitrocellulose or methanol-activated 0.2-μm polyvinylidene difluoride membranes (GE Healthcare). Membranes were blocked with 5% nonfat milk in PBS–0.05% Tween 20 (Sigma-Aldrich) and then incubated with the following primary antibodies at indicated dilutions: Anti-vSlfn polyclonal rabbit serum raised against vSlfn was provided by G. Karupiah (University of Tasmania, Australia) and used 1:250, the polyclonal rabbit anti-CrmD was previously described (*29*), and the anti-D8 polyclonal rabbit serum was a gift from D. Ulaeto (Defense Science and Technology Laboratory, UK) and used at 1:2000. Commercial antibodies included anti–Strep-Tag–HRP (horseradish peroxidase) (diluted 1:10,000, Iba), anti–β-actin AC15 (1:7000, Sigma-Aldrich), anti-IRF3 (1:1000, Abcam), anti–phosphorylated IRF3-Ser^386^ (1:1000, Abcam), anti–α-tubulin (1:10,000, Upstate Biotech), anti-TBK1 (1:5000, Abcam), anti–phosphorylated TBK1-Ser^172^ (1:5000, Abcam), anti-STING (1:1000, Cell Signaling Technology), anti–phosphorylated STING-Ser^366^ (1:1000, Cell Signaling Technology), anti-cGAS (1:1000, Cell Signaling Technology), anti-MAVS (1:2000, Enzo Life Sciences), and rabbit anti-FLAG (1:2000, Sigma-Aldrich). After extensive washing, horseradish peroxidase–conjugated (GE Healthcare) or IRDye-conjugated (LI-COR) secondary antibodies were added and membranes were processed for enhanced chemiluminescence or infrared scanning using an Odyssey imager (LI-COR Biosciences).

### Mice

Balb/c and C57BL/6 mice were purchased from Charles River. The IFNAR-deficient mice (*IFNAR*^−/−^*)* on a C57BL/6 genetic background were obtained from N. Sevilla and were bred and maintained in the animal facilities of Instituto Nacional de Investigación y Tecnología Agraria y Alimentaria, Spain. All mice used in experiments were 5- to 8-week-old females.

### Ethical statement

All animal experiments were performed with special efforts to minimize animal suffering, in compliance with national and international regulations, and were approved by the Ethical Review Board of Centro de Biología Molecular Severo Ochoa and Consejo Superior de Investigaciones Científicas under reference PROEX 025/16.

### Infection of mice

The infections of groups of 5 to 10 animals per condition were performed with sucrose-purified virus diluted in PBS containing 0.1% bovine serum albumin. Mice were anesthetized with isoflurane and infected by subcutaneous inoculation into the footpad with 10 μl of virus inoculum containing the indicated infectious doses. For intranasal infection of C57BL/6 mice, the infectious dose was 10^5^ pfu in 10 μl. When indicated, intravenous infection of Balb/c mice with ECTV was performed by injection of 50 μl of virus inoculum containing 5 × 10^3^ pfu into the tail vein. After infection, viral inocula used were back-titrated by plaque assay to verify the administered viral dose. Mice were housed in ventilated racks under biological safety level 3 containment facilities and monitored daily for survival and weight and scored for clinical signs of illness (scores ranging from 0 for healthy animals to 4 for severely diseased animals). Those animals exhibiting a weight loss greater than 20% of their initial weight, accompanied by a high clinical score, were euthanized. Where indicated, 10 μl of PBS alone or containing 140 μg of 2′,3′-cGAMP (Sigma-Aldrich) per animal was injected into the footpad 16 hours before ECTV subcutaneous inoculation in the same footpad. Determination of virus titers in organs at 5 dpi was performed as previously described (*28*).

### RNA sequencing

Two groups of five Balb/c mice per condition were footpad-inoculated with 10^3^ pfu of the indicated viruses or mock-infected, and samples of DPLN and spleen were aseptically removed at 5 dpi and conserved in RNAlater (Qiagen) at 4°C. Total RNA extraction was performed using the ReliaPrep RNA Tissue Miniprep System (Promega) following the manufacturer’s indications, and both quality and integrity of the RNA samples were assessed with the Agilent 2100 Bioanalyzer (Agilent Technologies). Duplicated cDNA libraries per condition (one per animal group) were constructed with TruSeq RNA Sample Prep Kit v2 Set A (Illumina) using equal amounts of total RNA from five animals per library, strictly following the manufacturer’s instructions. In the case of the lymph nodes, tissues were pooled before RNA extraction and two cDNA libraries were also generated per condition. Libraries were sequenced using TruSeq SBS Kit v3-HS (Illumina) on Illumina Hiseq 2000. The fastq files containing the reads, after quality assessment with package FastQC, were mapped to the mouse genome (build GRCm38 from *Mus musculus* C57BL/6J strain) using TopHat v2.0.4 mapping software with default parameters. Only those reads matching the mouse genome were considered in the differential gene expression analysis carried out with Cuffdiff (Cufflinks v2.1.0 software). Differentially expressed genes displaying statistically significant alterations (*P* < 0.005 and fold change > 2, using two replicates for condition) were used to determine those pathways that were affected by the viral infection. Pathway gene enrichment and upstream regulator analyses were performed with the Ingenuity Pathway Analysis software (Qiagen). Fastq files containing raw reads from RNA-seq can be accessed at ENA under project number PRJEB34111.

### Flow cytometry

Groups of six Balb/c mice per condition were mock-infected or infected with 10^3^ pfu of ECTV-WT or ECTVΔSlfn by subcutaneous inoculation in the footpad. DPLNs from each animal were collected at 2 dpi and pooled together in cold DMEM according to the same experimental condition. Three groups containing DPLNs from two animals each were processed. To obtain a cellular suspension, samples were incubated for 30 min at 37°C with collagenase IV (2 mg/ml) (Roche) and deoxyribonuclease I (200 U/ml) (Roche) and then passed through 40-μm cell strainers (BD Biosciences). After cell number estimation by manual counting in a hemocytometer, blocking of unspecific antibody binding sites was performed by incubation with CD16/CD32 Fc Block (BD Biosciences) before detection of cell surface antigens with monoclonal antibodies anti-CD3 (145-2C11) phycoerythrin (PE)–Cy7–conjugated and anti-CD49b (DX5) fluorescein isothiocyanate (FITC)–conjugated (BD Biosciences). To determine cell viability, cells were incubated with a LIVE/DEAD Fixable Near-IR Dead Cell Stain kit for 633- or 635-nm excitation (Molecular Probes). After fixation with a Fixation/Permeabilization kit (BD Biosciences), to detect intracellular granzyme B, cells were incubated with monoclonal antibody anti–granzyme B PE-conjugated (eBioscience). Data were acquired on a FACSCanto II flow cytometer (BD Biosciences) and analyzed using FlowJo software (Tree Star, USA). The gating strategy used is described in detail in fig. S6. The proportion of F4/80^+^ and CD11b^+^ cells from bone marrow–derived cells was estimated by flow cytometry after surface expression detection using anti-CD11b–APC (allophycocyanin) diluted 1:400 (BD Biosciences) and anti-F4/80–PE diluted 1:200 (BioLegend).

### Statistical analysis

Representative results from at least three independent repeats are shown for every cell biological assay. Data were analyzed using GraphPad Prism software. For comparisons between groups in cell biological assays, unpaired Student’s *t* test was applied. Survival data were analyzed using log rank (Mantel-Cox). Footpad swelling and % initial weight data were analyzed using multiple *t* tests with false discovery rate = 1%, while Mann-Whitney *U* test was used with data related to virus titers in organs. Analyses were performed up to times post-infection at which survival rates in the corresponding mice groups were above 50%.

## Supplementary Material

abb4565_SM.pdf

## SUPPLEMENTARY MATERIALS

Supplementary material for this article is available at http://advances.sciencemag.org/cgi/content/full/6/38/eabb4565/DC1

## Appendix

View/request a protocol for this paper from *Bio-protocol*.
