# Supplementary material for: Viral cGAMP nuclease reveals the essential role of DNA sensing in protection against acute lethal virus infection
Source: Sci Adv. 2020 Sep 18;6(38):eabb4565. doi: 10.1126/sciadv.abb4565 (PMC7500930; doi:10.1126/sciadv.abb4565)
Supplement: abb4565_SM.pdf [file abb4565_SM.pdf]

Science Advances
AAAS

advances.sciencemag.org/cgi/content/full/6/38/eabb4565/DC1

Supplementary Materials for

Viral cGAMP nuclease reveals the essential role of DNA sensing in protection against acute lethal virus infection

Bruno Hernáez, Graciela Alonso, Iliana Georgana, Misbah El-Jesr, Rocío Martín, Kathy H. Y. Shair, Cornelius Fischer, Sascha Sauer, Carlos Maluquer de Motes*, Antonio Alcamí*

*Corresponding author. Email: c.maluquerdemotes@surrey.ac.uk (C.M.d.M.); aalcami@cbm.csic.es (A.A.)

Published 18 September 2020, *Sci. Adv.* **6**, eabb4565 (2020)
DOI: 10.1126/sciadv.abb4565

This PDF file includes:

Figs. S1 to S6
Tables S1 and S2

Supplementary Materials

Activation of DNA sensing is essential for protection against acute lethal virus infection.

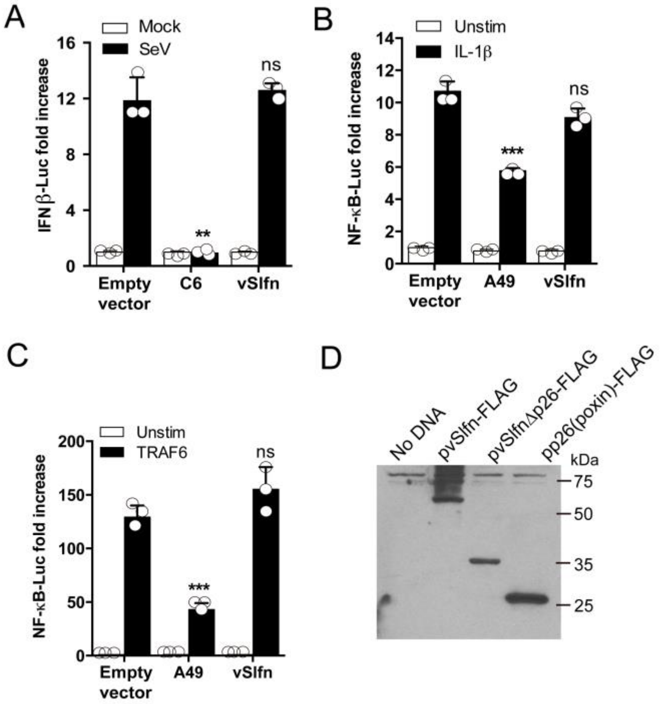

Fig. S1. vSlfn does not inhibit RNA virus-induced IFNβ or NF-κB reporter activation. (A-C) HEK293T cells expressing firefly luciferase under the control of the IFNβ (A) or NF-κB (B-C) promoters were transfected with vectors encoding vSlfn or VACV virus proteins C6 and A49, or the corresponding empty vector as controls. C6 and A49 are known inhibitors of IRF3 and NF-κB signaling included here as controls. (A) 24 h post-transfection, cells were infected or not with Sendai virus [1] and luciferase activity measured at 24 hpi. (B) Similarly, cells were stimulated or not with interleukin (IL)-1β for 8 h before luciferase activity determination. (C) Cells were transfected as above and simultaneously transfected with TNF receptor associated factor 6 (TRAF6). After 16 h, luciferase activity was determined. In all cases, luciferase activity was measured, normalized and presented as a fold increase over unstimulated EV-transfected cells. Data are presented as mean ± SD and represent one of at least three experiments performed in triplicate. ***, $p < 0.001$ (unpaired Student's *t* test comparing with empty vector). (D) Expression of FLAG-tagged versions of vSlfn. HEK293T cells were independently transfected with expression vectors encoding full-length vSlfn, vSlfn lacking p26 domain (vSlfnΔp26) or p26 domain, fused with a C-terminal 3xFLAG epitope. Whole cell lysates were analyzed 24 h after transfection by SDS-PAGE and immunoblotting using an antibody against FLAG.

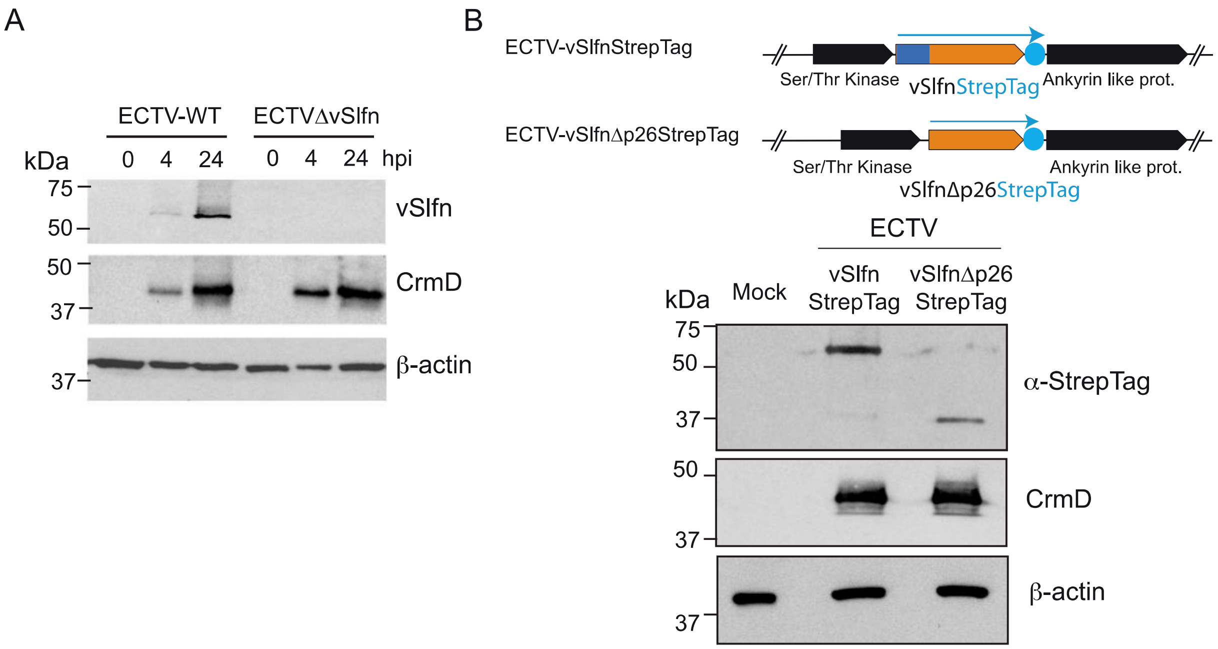

Fig. S2. Absence of vSlfn expression in ECTVΔvSlfn infected cells. (A) Analysis of vSlfn expression by western blot in BSC-1 cells after infection with 5 pfu/cell of ECTV-WT and ECTVΔvSlfn at the indicated times post-infection. (B) Analysis of vSlfn and vSlfnΔp26 expression from recombinant ECTVs expressing C-terminally Strep-Tag tagged versions of vSlfnΔp26 and full length vSlfn (ECTV-vSlfnΔp26StrepTag and ECTV-vSlfnStrepTag, respectively). BSC-1 cells were infected as above and lysates analyzed by immunoblotting using an anti-StrepTag antibody at 24 hpi, confirming expression of vSlfnΔp26. ECTV protein CrmD was used as control of infection and β-actin as protein loading control. One representative experiment from two performed is shown. Representation of the genomic organization of vSlfn locus and surrounding loci in the recombinant viruses used is shown.

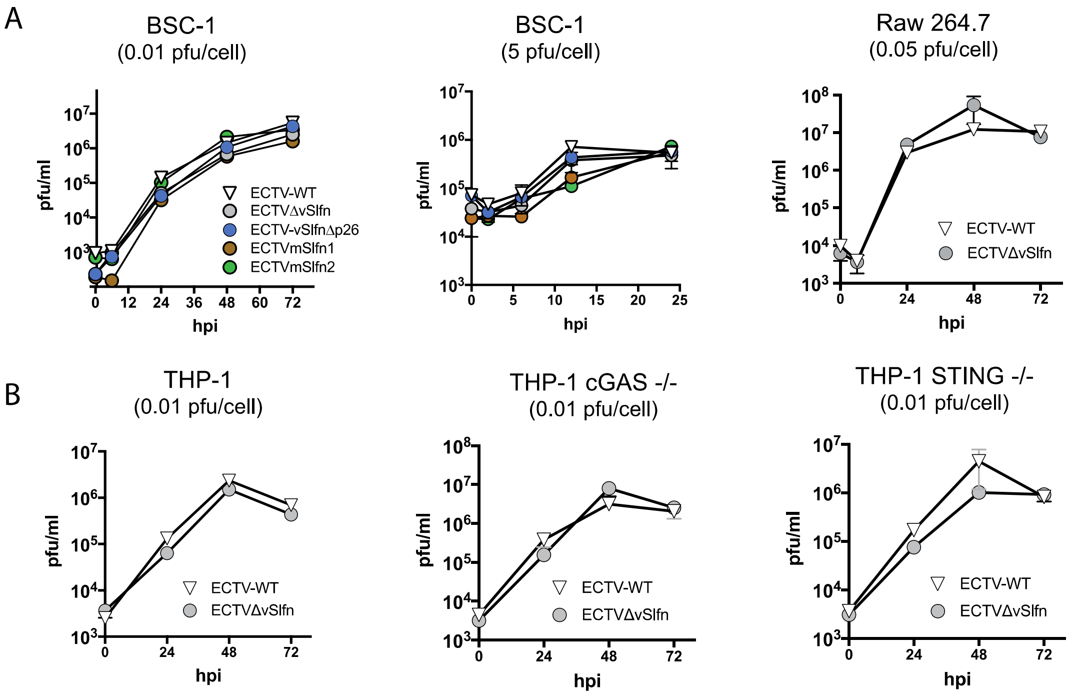

Fig. S3. Absence of vSlfn in ECTVΔvSlfn does not affect viral replication. A) To analyze ECTV replication diverse types of cells were infected with the indicated viruses. Results showed that all recombinant viruses had similar kinetics of replication *in vitro* to ECTV-WT. B) Similarly, ECTV replication in the presence or absence of vSlfn was analyzed in THP-1 cells and THP-1 cells lacking STING or cGAS expression. The multiplicity of infection of the viral inoculum is indicated in parenthesis. BMDM, Bone marrow derived macrophages.

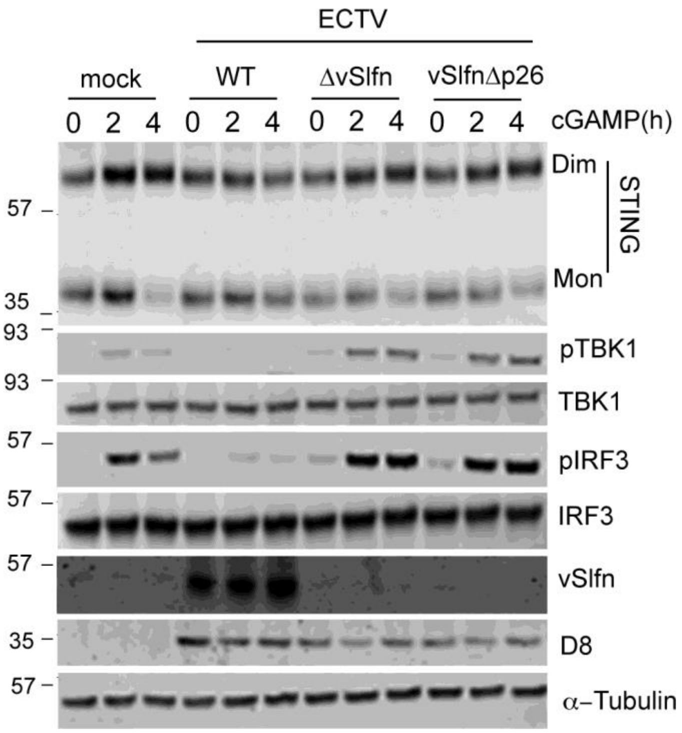

Fig. S4. vSlfn prevents STING activation in response to cGAMP during ECTV infection. PMA-differentiated THP-1 cells were infected with 2 pfu/cell of the indicated viruses for 6 h before stimulation with cGAMP. Cell lysates were harvested at indicated times after cGAMP addition and analyzed by SDS-PAGE and immunoblotting with antibodies against the indicated proteins. The levels of cGAMP-induced STING dimerization as well as TBK1 and IRF3 phosphorylation were reduced during ECTV-WT infection, but remained similar to those in mock-infected cells during ECTVΔvSlfn and ECTV-vSlfnΔp26 infection. D8 is an unrelated ECTV protein used as infection marker.

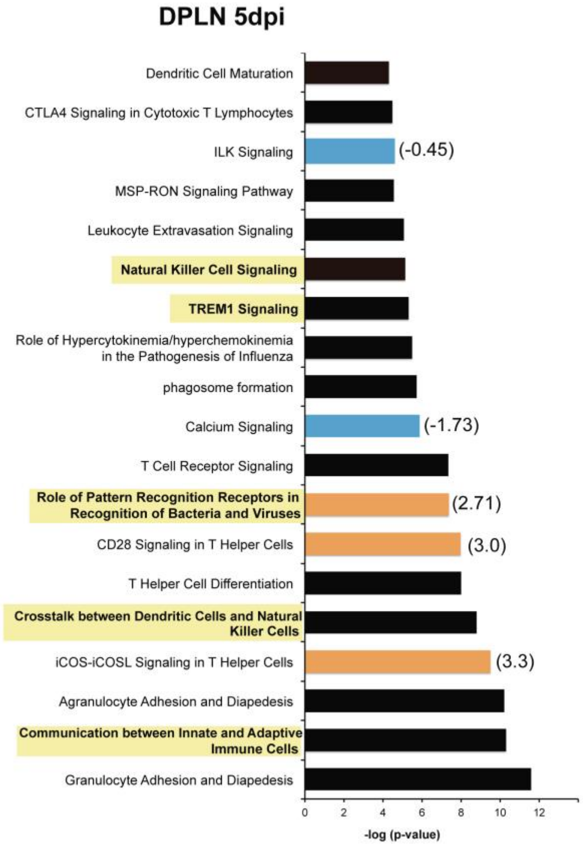

Fig. S5. Pathway enrichment analysis from DPLNs at 5 dpi. Similar to Fig. 4., DPLNs from ECTV-WT and ECTVΔvSlfn infected animals were collected at 5 dpi for RNA-sequencing and further differential expression analysis to compare wild type with ECTVΔvSlfn infection. Analysis of the corresponding differentially expressed genes revealed the enrichment of diverse pathways related to innate immunity (highlighted). Z-score values for activated (orange) or repressed (blue) predicted pathways are indicated.

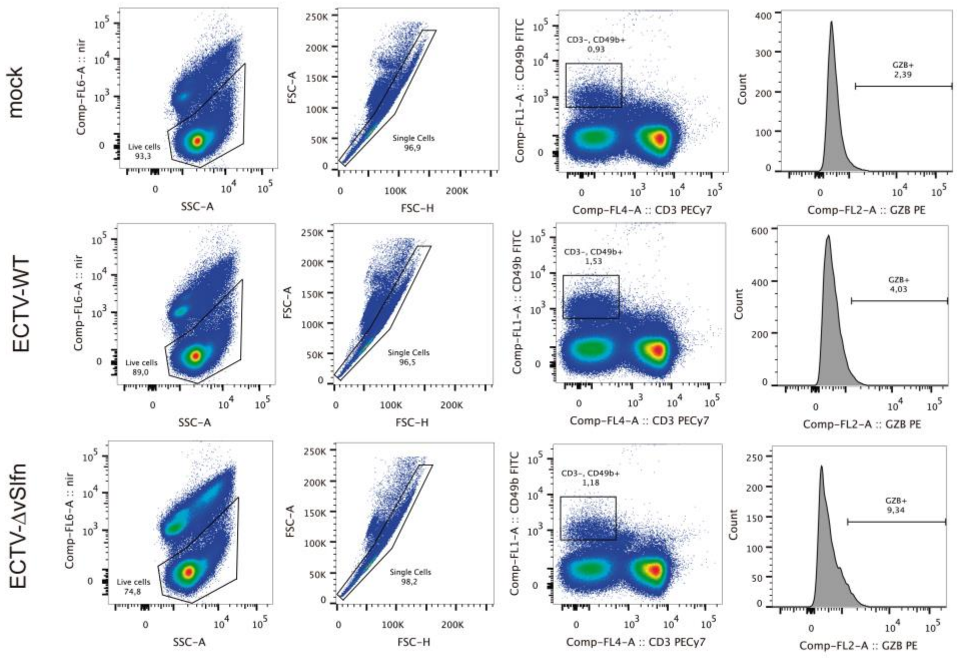

Fig. S6. Gating strategy for data in Fig. 6 and a representative example of each experimental condition are shown. The numbers of events positively stained in corresponding fluorescence minus one control samples were 0.069 % and 0.001 % positives in the case of CD49b and granzyme b, respectively.

**Dose (pfu/animal)**	**ECTV**							
	**WT**	**ΔvSlfn**		**vSlfnΔp26**	**mSlfn1**		**mSlfn2**	
1	2/5	n.d.	n.d.	n.d.	n.d.	n.d.	n.d.	n.d.
10	0/5	5/5	n.d.	n.d.	n.d.	n.d.	n.d.	n.d.
10_2	n.d.	3/5	n.d.	5/5	n.d.	n.d.	n.d.	n.d.
10_3	n.d.	5/5	5/5	5/5	5/5	4/5	5/5	5/5
10_4	n.d.	n.d.	5/5	5/5	5/5	n.d.	5/5	n.d.
10_5	n.d.	n.d.	5/5	5/5	5/5	n.d.	5/5	n.d.
10_6	n.d.	n.d.	4/5	4/5	5/5	n.d.	4/5	n.d.

Table S1. Mortality rates of vSlfn recombinant ECTVs. Groups of 5 BALB/c mice were s.c. infected in the footpad with increasing doses of the indicated viruses and the number of survivors at 21 dpi determined for each condition are shown. Each column corresponds to an independent experiment. n.d., not determined.

Recombinant virus	Parental virus	Vector	5´flanking region oligos (5´-3´)	3´flanking region oligos (5´-3´)	Insert between Flanking Regions-oligos (5´-3´)
**ECTVΔvSlfn**	ECTV Naval strain	p33	GGAATTCATCAGATGGATAAGAATA	GGGATCCGTTGTGCTGTGTTTGCAG	
			GGGATCCGTTTTCGGTTGCACTCGC	GGCTGCAGCTAGATAAAAAATCAACT	
**ECTV-vSlfnΔp26**	ECTV Naval strain	pBH4		GCGCGGATCCATGAAAGGTGCGGTGCTACA	
				GGCTGCAGCTAGATAAAAAATCAACT	
**ECTV-mSlfn1**	ECTV Naval strain	p35	GGAATTCATCAGATGGATAAGAATA	GGGATCCGTTGTGCTGTGTTTGCAG	CGAGCTCATAATGAACATCACCGAT
			GGGATCCGTTTTCGGTTGCACTCGC	GGCTGCAGCTAGATAAAAAATCAACT	CGAGCTCCTAAGACATGAGGAGCTT
**ECTV-mSlfn2**	ECTV Naval strain	pBH2	GGAATTCATCAGATGGATAAGAATA	GGGATCCGTTGTGCTGTGTTTGCAG	CGGATCCATGGGTACTAGACTTGAGGC
			GGGATCCGTTTTCGGTTGCACTCGC	GGCTGCAGCTAGATAAAAAATCAACT	CGGATCCTCAACCTGATGGGGCATTCATC
**ECTV-vSlfnΔp26StrepTag**	ECTV-vSlfnΔp26	pBH5	GCGGAATTCCGGCGGTGGCACAATCGAGG	GCGGGATCCTTAATGTAACTATAGAG	
			GCGGGATCCTCACTTCTCGAATTGAGGGTG	GCGCTGCAGTGGATGATAACCCGAGATTA	
**ECTV-vSlfnStrepTag**	ECTV Naval strain	pBH5	GCGGAATTCCGGCGGTGGCACAATCGAGG	GCGGGATCCTTAATGTAACTATAGAG	CGGATCCATGGGTACTAGACTTGAGGC
			GCGGGATCCTCACTTCTCGAATTGAGGGTG	GCGCTGCAGTGGATGATAACCCGAGATTA	CGGATCCTCAACCTGATGGGGCATTCATC

Expression Vector	Description	Oligos for InFusion cloning
**pvSlfn-FLAG (pBH21)**	Expresses FLAG-tagged vSlfn optimized for expression in mammalian cells	n/a
**pvSlfnΔp26-FLAG (pBH32)**	Expresses FLAG-tagged vSlfn lacking p26 optimized for expression in mammalian cells	GCGGCCGCGCCACCATGAAAGGCGCCGTGCTGCAGATCCCCAA
		CTGCAGCACGGCGCCTTTCATGGTGGCGCGGCCGCTCGAG
**pp26-FLAG (pBH33)**	Expresses FLAG-tagged vSlfn p26 domain optimized for expression in mammalian cells	CTGCAGATCCCCAACGTGTCCTACATCAAAGTGATCGATGAC
		TTTGATGTAGGACACGTTGGGGATCTGCAGCACGGCGCCTTT

Table S2. Vectors generated in the present work. Oligonucleotides used to generate the recombination vector together with the parental virus used in the construction of each recombinant mutant virus are detailed. Every recombination vector is based on previous pMS30. Below, mammalian expression vectors generated in this study and used in the reporter gene assays are shown.
